# Supplementary material for: Modelling Skylarks (Alauda arvensis) to Predict Impacts of Changes in Land Management and Policy: Development and Testing of an Agent-Based Model
Source: PLoS One. 2013 Jun 6;8(6):e65803. doi: 10.1371/journal.pone.0065803 (PMC3675089; doi:10.1371/journal.pone.0065803)
Supplement: Supporting Information S4 — The skylark ODdox as a zipped archive. (ZIP) [file pone.0065803.s004.zip › Skylark_ODdox/class_movement_map16-members.html]

ALMaSS Skylark ODdox: Member List


ALMaSS Skylark ODdox
 2.0


- Main Page
- Related Pages
- Classes
- Files

- Class List
- Class Index
- Class Hierarchy
- Class Members

MovementMap16 Member List

This is the complete list of members for MovementMap16, including all inherited members.

|  |  |  |
| --- | --- | --- |
| ClearMapValue(unsigned x, unsigned y) | MovementMap16 |  |
| GetMapValue(unsigned x, unsigned y) | MovementMap16 |  |
| Init() | MovementMap16 | protected |
| m\_ALandscape | MovementMap16 | protected |
| m\_TheMap | MovementMap16 |  |
| maxx | MovementMap16 |  |
| maxy | MovementMap16 |  |
| MovementMap16(Landscape \*L) | MovementMap16 |  |
| SetMapValue(unsigned x, unsigned y, unsigned value) | MovementMap16 |  |
| ~MovementMap16() | MovementMap16 |  |


- Generated on Thu Jan 10 2013 13:15:36 for ALMaSS Skylark ODdox by
   1.8.1.1
